# 

**Published:** 1892-04-16

**Authors:** 


### CONTENTS.

The Hospital Problem . . . . . . . . . . 33

Passing Topics.—Saturday Fund Statistics . . . . . 34

The Doctor's Armchair.—Impatient Science . . . . 35

Home Libraries in Boston . . . . . . . . 36

Medical History from the Earliest Times.—By E. T. Withington, M.A., M.B. Oxon. IV.—Medicine in Ancient Egypt . . . . . . . . . . . . 37

Everybody's Page . . . . . . . . . . 38

Annotations.—An Essex Arcadia.—The Sewage of Isolated Dwellings.—Galen's Diagnosis of Love Fever . . . . . 39

Notes and News, 40; Scraps and Gleanings . . . . 41

The Student of Medicine.—Appointments—Vacancies—Notes from the Schools—Pass Lists.

The Practitioner's Mirror and Retrospect—

Surgery.—Laminectomy for Compression Paraplegia Due to Spinal Caries, 43; Injuries to the Fingers . . . . 43

Ophthalmology.—Epiphora . . . . . . . . . 44

Practitioner's Bookshelf . . . . . . . . . 44

New Drugs, Appliances, and Things Medical.—Malt Pastilles.—Æsculap Water . . . . . . . . . 44

The Institutional Workshop—

The Chronic Indigence of our Hospitals, with some Suggestions for its Remedy. By B. Burford Rawlings. III.—The Material Deficit . . . . . . . . 45

Hospital Construction.—Asylum for the Insane, Waterbury, Vermont, 47; Hospital in Hong Kong . . . . . 48

### EXTRA SUPPLEMENT.—THE NURSING MIRROR.

En Passant.—Stockholm Nurses—Twenty Years Ago—How to Make Money—Sympathy—The Registration of Nurses—"Puffs"—East-end Mothers' Home . . . . . xiii

Ventilation, Disinfection, and Diet. By P. Caldwell Smith, M.D. . . . . . . . . . . xiv

Asylum Attendants . . . . . . . . . . xv

For Reading to the Sick.—The Sea . . . . . . . xv

On the Nursing of Children. VIII.—Clothes, Food, &c. . xvi

The Old and the New . . . . . . . . . . xvii

Hints to Nurses.—Marriage.—Appointment . . . . xvii

Everybody's Opinion—Talking Shop—Mrs. Creighton Hale's New Book on Massage.—The Nurses' Co-operation.—Holiday Experiences . . . . . . . . . . . xviii

Greetings from Vancouver—Notes and Queries . . . xviii

Among the Institutions . . . . . . . . . xix

On Board the Antwerp Boat . . . . . . . . xix

